# Remodeling adipose tissue inflammasome for type 2 diabetes mellitus treatment: Current perspective and translational strategies

**DOI:** 10.1002/btm2.10150

**Published:** 2019-12-13

**Authors:** Amrita Banerjee, Jagdish Singh

**Affiliations:** ^1^ Department of Pharmaceutical Sciences North Dakota State University Fargo North Dakota

**Keywords:** adipokines, chronic inflammation, cytokines, formulations, obesity, type 2 diabetes mellitus

## Abstract

Obesity‐associated type 2 diabetes mellitus (T2DM) is characterized by low‐grade chronic systemic inflammation that arises primarily from the white adipose tissue. The interplay between various adipose tissue‐derived chemokines drives insulin resistance in T2DM and has therefore become a subject of rigorous investigation. The adipocytokines strongly associated with glucose homeostasis include tumor necrosis factor‐α, various interleukins, monocyte chemoattractant protein‐1, adiponectin, and leptin, among others. Remodeling the adipose tissue inflammasome in obesity‐associated T2DM is likely to treat the underlying cause of the disease and bring significant therapeutic benefit. Various strategies have been adopted or are being investigated to modulate the serum/tissue levels of pro‐ and anti‐inflammatory adipocytokines to improve glucose homeostasis in T2DM. These include use of small molecule agonists/inhibitors, mimetics, antibodies, gene therapy, and other novel formulations. Here, we discuss adipocytokines that are strongly associated with insulin activity and therapies that are under investigation for modulation of their levels in the treatment of T2DM.

## 1 INTRODUCTION

Type 2 diabetes mellitus (T2DM) is currently considered a global epidemic and projected to rise exponentially by more than 50% worldwide in the next 20 years.^1^, ^2^ Particularly, few central African and middle‐eastern countries will see a projected more than 100% rise in diabetes cases from 2015 to 2040.^1^ Life style changes in the modern era, food habits, and environmental pollutants are primarily responsible for the rapid global rise in T2DM cases. Additionally, in developing or low‐income countries where infectious diseases present a more pressing problem, there can be lack of proper resources and/or knowledge among primary caregivers for management of this slow but silent killer. According to a World Health Organization report, there were an estimated 1.6 million fatalities in 2016 due to the disease, making diabetes the seventh leading cause of deaths and the major cause of blindness, renal failure, cardiac arrests, stroke, and amputations of the lower limbs.^3^ Given these alarming facts, it is paramount to develop efficient therapies that not only halt the progression but also treat the underlying cause of the disease.

T2DM occurs primarily due to insulin resistance in adipose tissue, muscles, and liver leading to hyperglycemia. Obesity, age, family history of diabetes, hypertension, hypercholesterolemia, and presence of cardiovascular diseases are considered some of the biggest risk factors for T2DM.^4^ Therefore, in addition to diabetes pharmacotherapy, care often requires management of the aforementioned co‐conspirator diseases, consumption of healthy foods, and regular exercise to alleviate diabetic conditions. Among the various antidiabetic medications, the most commonly prescribed class of medications include biguanides (metformin), glucagon‐like peptide‐1 agonists/receptor agonists, sodium glucose transport protein‐2 inhibitors, dipeptidyl peptidase 4 inhibitors, thiazolidinediones (TZDs), sulfonylureas, and insulin.^5^

**Figure 1:**
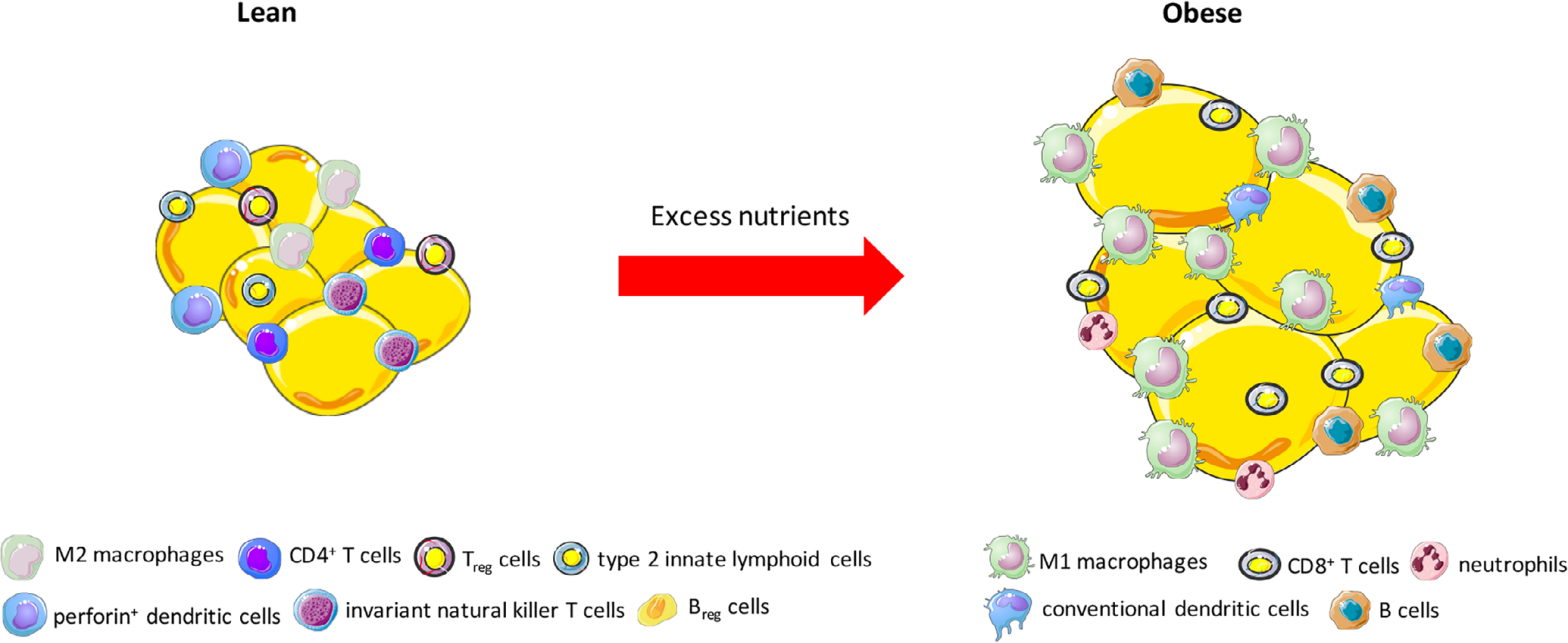
Adipose tissue modulation in obesity. Adipose tissue hypertrophy in obesity results in significant immunomodulation that skews adipose tissue microenvironment toward proinflammatory phenotype. Several immune cells normally present in adipose tissue of lean individuals are downregulated in obesity (not shown in the figure). For more details about immune cell changes in adipose tissue with obesity and type 2 diabetes, refer to publication by Lu et al^10^

In obesity, adipocyte hypertrophy can cause secretion of proinflammatory adipokines such as monocyte chemoattractant protein 1 (MCP‐1), which recruits circulating monocytes from the blood circulation to the adipose tissue, where they undergo differentiation into macrophages that are predominantly polarized to proinflammatory M1 phenotype.^6^, ^7^ Over time, the white adipose tissue of obese individuals can comprise up to 40–50% macrophages that are M1 polarized, which is in clear contrast to lean individuals who have only about 5% macrophages in their adipose tissue and they are primarily polarized to noninflammatory M2 phenotype.^8^ The M1 macrophages secrete proinflammatory cytokines into the systemic circulation such as tumor necrosis factor‐α (TNF‐α), interleukin 1β (IL‐1β), IL‐6, IL‐18, and C‐X‐C motif chemokine 5 (CXCL5), among others.^9^ Additionally, dysregulation of other immune cells in the adipose tissue such as T cells, B cells, and dendritic cells is observed in obesity‐associated T2DM. An increased accumulation of conventional dendritic cells, CD8^+^ cytotoxic T lymphocytes, and proinflammatory B cells in the adipose tissue exacerbates inflammation and insulin resistance.^10^ Conversely, population of M2 macrophages, regulatory B or T cells, type 2 innate lymphoid cells, eosinophils, perforin^+^ dendritic cells, and invariant natural killer T cells that are responsible for regulation of immunity and metabolic homeostasis in the adipose tissue are substantially reduced in obesity^11^ (Figure 1).

**Figure 2:**
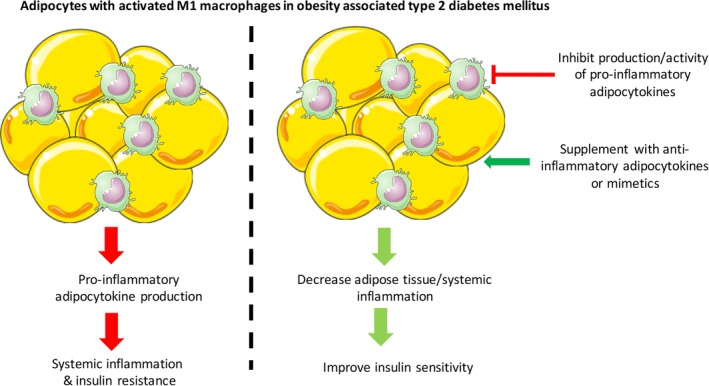
Adipose tissue inflammation in obesity‐associated type 2 diabetes mellitus and its treatment perspective. For simplicity only M1 macrophages are shown in the figure with the adipocytes

Likewise, the adipocytes in obese individuals secrete several adipokines implicated in insulin resistance such as resistin, retinol‐binding protein 4 (RBP‐4), MCP‐1, progranulin, chemerin, and angiopoietin‐like protein 2 (ANGPTL2).^9^, ^12^ Similar pathophysiology is observed in other key insulin target organs such as muscles and liver. In muscles, increased accumulation of extramyocellular adipocytes lead to macrophage recruitment and activation, while in liver, the resident macrophage Kupffer cells are activated, which increase inflammatory cytokine production.^13^ Chronic presence of these inflammatory adipocytokines is widely implicated to cause insulin resistance in insulin‐sensitive organs, resulting in T2DM development.^14^, ^15^, ^16^, ^17^ A concomitant decrease in insulin‐sensitizing adipokines (adiponectin, leptin, apelin, omentin, and secreted frizzled protein‐5) and anti‐inflammatory cytokines (IL‐10 and IL‐22) is also observed, which exacerbates inflammatory conditions and subsequent insulin resistance.^12^, ^18^ Therefore, remodeling of adipose tissue inflammasome through modulation of adipocytokine production is a rational step toward efficient treatment of obesity‐associated T2DM (Figure 2).

Many antidiabetic medications‐based therapies are symptomatic and do not address the underlying cause of the diseases such as chronic inflammation in the white adipose tissue of obese/overweight individuals. Metformin was recently shown to decrease adipose tissue inflammation while TZDs have been reported to decrease adipose tissue macrophage content and improve insulin sensitivity.^19^, ^20^, ^21^ TZDs are however associated with several significant risks including heart failure, bladder cancer, diabetic macular edema, among others.^22^, ^23^, ^24^ Anti‐inflammatory therapies that target adipose tissue inflammation can significantly improve insulin sensitivity and β‐cell function, and therefore have great potential in the treatment of T2DM.^25^, ^26^ This review discusses some of the major adipocytokines involved in T2DM development and few notable formulations/drugs that are being developed to halt the vicious cycle of chronic inflammation in the adipose tissue and development of systemic insulin resistance.

## 2 ADIPOKINES AND THERAPIES THAT MODULATE THEIR ACTIVITY

**Figure 3:**
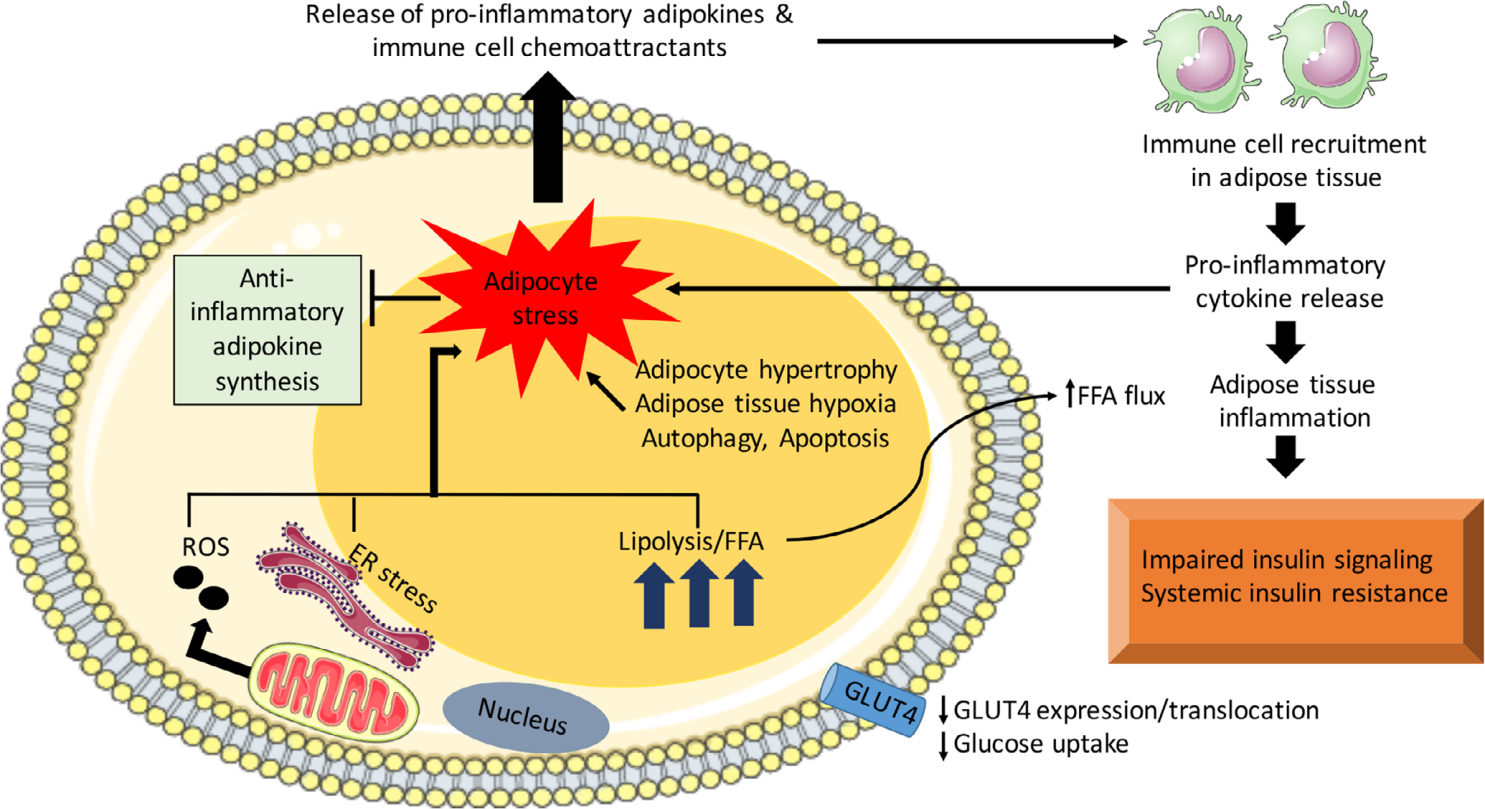
Mechanism of inflammation activation in an adipocyte leading to insulin resistance. Adipocyte stress due to hypertrophy, adipose tissue hypoxia, autophagy, apoptosis, ROS (reactive oxygen species) production, ER (endoplasmic reticulum) stress, lipolysis leading to production, and increased flux of FFA (free fatty acids) result in secretion of proinflammatory adipokines and downregulation of anti‐inflammatory adipokines.^27^ The proinflammatory adipokines recruit immune cells in the adipose tissue that differentiate to proinflammatory phenotype and release proinflammatory cytokines that further promulgate inflammatory conditions. Chronic inflammatory conditions and high serum FFA concentration lead to impairment in insulin signaling and systemic insulin resistance. A decrease in GLUT4 (glucose transporter 4) expression and translocation to the cell membrane causing reduction in glucose uptake and glucose toxicity also occurs. For more details on mechanisms of adipose tissue inflammation and insulin resistance, refer to publication by Bluher^27^

Adipocyte hypertrophy can cause secretion of proinflammatory adipokines and initiation of adipose tissue inflammation. The various mechanisms involved in activation of inflammation in adipocytes leading to insulin resistance are presented in Figure 3. These molecules have therefore garnered immense interest among researchers who have attempted to either increase serum levels of insulin‐sensitizing adipokines or decrease concentration of insulin resistance imparting adipokines. Major adipokines involved in glucose homeostasis and notable drugs/formulations under investigation for modulation of their activities are discussed in this section.

### 2.1 Adipokines mediating insulin sensitization

Among various adipokines, adiponectin secreted exclusively by adipocytes plays a key role in glucose and lipid metabolism in insulin‐sensitive tissues and was shown to decrease blood glucose levels in many preclinical studies performed on diabetic animals. It improves insulin sensitivity and free fatty acid oxidation while decreases hepatic glucose output and vascular inflammation.^28^ In a recent study, higher adiponectin level was associated with decreased T2DM risk in Chinese and other populations.^29^ Proinflammatory cytokines, especially TNF‐α, are known to reduce adiponectin levels.^30^ A low serum adiponectin level is strongly associated with obesity and development of insulin resistance with a concurrent downregulation of adiponectin receptor expression.^31^, ^32^ Conversely, adiponectin administration leads to improvement in insulin sensitivity, decrease in plasma glucose levels, and reduced MCP‐1 production.^33^, ^34^, ^35^ Adiponectin formulations mainly include administration of recombinant adiponectin produced and isolated from mammalian or *Escherichia coli* cultures after transfection of plasmid encoding adiponectin. A single injection of recombinant adiponectin was found to transiently decrease hyperglycemia in *ob/ob*, NOD, and streptozotocin‐induced diabetic mice.^36^ In a different study, administration of globular head domain of adiponectin decreased free fatty acids in the serum and was postulated to be due to increased oxidation of fats by muscle.^37^ Similarly, intraperitoneal administration of recombinant adiponectin decreased visceral adiposity and weight gain in agouti yellow obese mice.^38^ However, Novo Nordisk obtained contradictory preclinical evidence on recombinant adiponectin therapy in lowering blood glucose levels in T2DM.^39^ The research group showed that intraperitoneal injection of human and murine adiponectin produced from CHOK1SV and HEK293 cells respectively were ineffective in reducing plasma glucose levels in *db/db* diabetic mice or sand rats. They posit that adiponectin may require processing by adipocytes to demonstrate bioactivity. In light of this contradictory evidence, researchers have successfully developed an orally bioavailable adiponectin mimetic small molecule drug, AdipoRon, that strongly binds to adiponectin receptors (AdipoR1 and AdipoR2) and ameliorates diabetic conditions while extending lifespan in diabetic *db/db* mice placed on high‐fat diet.^40^ Gene therapy for adiponectin supplementation has also been extensively studied for diabetes treatment. For example, when adiponectin cDNA subcloned in adenoviral vector containing a muscle specific promoter was delivered through electroporation and intramuscular injection, it resulted in significant improvement in insulin sensitivity in mice receiving high‐fat–high‐sucrose diet.^41^ Similarly, nonviral‐based adiponectin gene delivery using mini‐circle DNA in polyethyleneimine carrier resulted in optimum serum adiponectin levels and normalization of parameters pertaining to insulin resistance in obese C57BL/6J mice.^42^ Intravenous administration of adiponectin pDNA in streptozotocin‐induced diabetic mice resulted in 10–15‐fold enhancement in adiponectin serum levels as well as hepatic glucose uptake that led to reduction in serum glucose and triglyceride levels.^43^

Leptin is another important adipokine that acts through its receptor in the brain and is involved in glucose homeostasis, regulation of energy expenditure, and appetite.^44^ Serum levels of leptin are very low in obesity with leptin deficiency, lipodystrophy‐induced T2DM, HIV‐lipodystrophy‐induced T2DM, and in type 1 diabetes mellitus (T1DM) with lipodystrophy.^45^ In patients presenting these cases, leptin intervention can markedly improve glucose and lipid homeostasis.^45^ However, leptin levels are high in generalized obesity or obesity‐associated T2DM and such patients show poor efficacy to exogenous leptin administration.^45^ This may be attributed to leptin resistance in common obesity, which is mostly caused by disruption in leptin signaling, impairment in regulation of feeding/reward behavior, or reduced leptin transport across the blood–brain barrier (BBB).^46^ A synthetic human leptin analog, Metreleptin (Myalept™), has been FDA approved for treatment of lipodystrophy and complications arising due to leptin deficiency. To improve leptin delivery to the brain, intrathecal leptin administration to bypass the BBB, polyethylene glycol (PEG)‐grafted leptin to increase the circulation half‐life of the protein, oil‐based subcutaneous injection of leptin for slower release, and prolonged activity or leptin peptide agonists have been investigated, which demonstrated varying success.^47^ Adjuvant therapy using epinephrine and norepinephrine was found to modulate leptin receptor activity and significantly improve leptin transport.^47^ Gene delivery through intracranial injection to hypothalamus using adenoviral vector encoding human leptin to *ob/ob* mice led to body weight reduction in 4 weeks.^48^ Similarly, intraventricular administration of adenovirus encoding rat leptin DNA led to 17% lower weight and 80% lesser white adipose tissue in female rats compared to sham control.^49^ Intranasal delivery of pluronic P85 conjugated to N‐terminal portion of leptin led to higher accumulation in hippocampus and hypothalamus than native leptin administered intranasally.^50^ This resulted in activation of leptin receptors in hypothalamus at a lower dose than unmodified leptin. This technology is now being developed by NeuroNanoPharma Inc. (Raleigh, NC) for treatment of obesity.

Apelin, an adipokine that is also expressed in the brain, is postulated to possess anti‐inflammatory properties through inhibition of reactive oxygen species (ROS).^51^ Its expression in adipocytes increases with increase in insulin concentration such as in obesity–hyperinsulinemia animal models and upon insulin treatment in cultured adipocytes in vitro.^51^ Increase in hypoxia in adipose tissue that promulgates inflammatory conditions also induces apelin production.^51^ Existence of apelinemia (high apelin concentration) has been found in morbidly obese and patients without morbid obesity but exhibiting impaired glucose tolerance or T2DM.^52^ However, there are contradictions to the findings as apelin levels were found lower in obese T2DM patients not treated with antidiabetic drugs such as metformin and rosiglitazone.^52^ In the same vein, no correlation has been found between apelin levels and body weight, adiposity and insulin resistance in obese and lean children.^53^ An insulin‐sensitizing/insulin‐mimetic effect of apelin has been postulated after studies in high‐fat‐diet‐induced obese T2DM mice, where intravenous apelin injection significantly improved glucose tolerance and insulin sensitivity.^54^ Additionally, peripheral apelin administration was found to improve skeletal muscle glucose utilization, decrease triglycerides, free fatty acids, adiposity, body weight, and insulinemia.^51^ Two enzyme degradation‐resistant acylated analogs of apelin‐13 amide, (Lys^8^GluPAL)apelin‐13 amide and pGlu(Lys^8^GluPAL)apelin‐13 amide, were found to attenuate diabetic conditions, improve weight loss, and decrease circulating triglycerides and LDL cholesterol while increasing HDL cholesterol in high‐fat‐diet‐fed mice compared to saline control.^55^ A clinical trial on the influence of pyr1‐apelin‐13 (exopeptidase degradation resistant) on insulin sensitivity in healthy overweight men has been completed and shown to significantly improve insulin sensitivity in a hyperinsulinemic–euglycemic clamp study as well as cause no adverse effects due to apelin administration.^56^ A long circulating PEGylated variant of apelin‐36 was found to significantly lower blood glucose and improve glucose tolerance in diet‐induced obese mice.^57^

Omentin‐1 is a novel adipokine identified from omental fat pad and is preferentially secreted by the visceral rather than subcutaneous adipose tissue.^58^ It has anti‐inflammatory, anti‐atherosclerotic, and cardioprotective properties.^59^ In addition, it enhances insulin signal transduction, regulates blood glucose via insulin‐mediated glucose uptake in adipocytes, and is involved in lipid metabolism.^60^, ^61^ Compared to healthy individuals, the serum level of omentin‐1 is significantly lower in conditions that display insulin resistance such as in obesity, T2DM, gestational diabetes, and in women with polycystic ovarian syndrome, but increases with weight reduction.^61^, ^62^ However, absence of efficacy in basal glucose uptake suggests that omentin‐1 lacks inherent insulin‐like activity.^60^ When rat primary cardiomyocytes exposed to conditioned media from epicardial adipose tissue of T2DM patients were treated with recombinant omentin‐1, contractile dysfunction and insulin resistance were prevented, demonstrating a cardioprotective role of the adipokine in T2DM.^63^ However, omentin‐1 is not being actively pursued as a therapy for T2DM in any current clinical trial.

Another novel adipokine C1q/TNF‐related protein‐3 (CTRP3, also called cartonectin) is considered an adiponectin paralog and was shown to be involved in glucose homeostasis.^64^ Administration of recombinant CTRP3 protein resulted in blood glucose lowering in normal and insulin‐resistant *ob/ob* mice, without affecting the levels of adiponectin or insulin. In human, circulating CTRP3 level is lower in obesity and negatively correlated with insulin resistance.^65^ Glucagon‐like peptide‐1 receptor agonist Exendin‐4 was shown to increase CTRP3 expression in vitro.^66^ Metformin treatment was also found to elevate serum CTRP3 levels in women with polycystic ovarian disease.^67^

Secreted frizzled protein‐5 (SFP5) is secreted from various tissues including visceral, subcutaneous, pericardial adipose tissues, liver, heart, pancreatic islets, and mononuclear blood cells.^68^ Different cross‐sectional clinical studies have found that SFP5 serum levels are inversely related to diabetes and cardiovascular disease risk factors, and its concentration is lower in prediabetes or T2DM.^68^, ^69^ Treatment with Liraglutide increased SFP5 levels.^69^ However, confounding results have been presented in other studies where investigators either found no significant difference in serum SFP5 levels between obese and nonobese individuals or SFP5 overexpression decreased glucose metabolism, with the latter tested only in preclinical trials.^70^, ^71^

Vaspin, also known as visceral adipose tissue‐derived serine protease inhibitor or serpin A12, is an insulin‐sensitizing adipokine.^72^, ^73^ Recombinant vaspin administration to obese mice was shown to improve glucose tolerance as well as insulin sensitivity and demonstrated to be mediated through inhibition of inflammation caused by TNF‐α, IL‐1, and other inflammatory modulators.^74^, ^75^ In contradiction to its role, vaspin concentration is elevated in obesity and T2DM, but this is generally considered a compensatory mechanism to impaired glucose homeostasis in obesity and T2DM.^75^ However, confounding results were documented in newly diagnosed T2DM patients where circulating vaspin levels were significantly diminished compared with age‐matched healthy controls, and a positive correlation between serum vaspin and insulin sensitivity was obtained.^76^ In corroboration, when overweight women with polycystic ovary syndrome were treated with metformin, vaspin levels decreased with concomitant improvement in insulin sensitivity.^77^

### 2.2 Adipokines mediating insulin resistance

Adipokines that stimulate insulin resistance include MCP‐1, RBP‐4, progranulin, chemerin, and ANGPTL2. These adipokines and formulations/strategies that inhibit their activity are discussed below.

MCP‐1 is implicated in migration and infiltration of monocytes into the adipose tissue that transition to inflammatory macrophages (M1 polarized). Inhibition of MCP‐1 using NOX‐E36 (ematicap pegol) for the treatment of T2DM and albuminuria has successfully completed phase IIa clinical trials and showed reduction in HbA1c levels in albuminuric T2DM patients.^78^ The drug is a Spiegelmer® (l‐isomers of RNA oligonucleotides)‐based therapeutic agent that interacts specifically with MCP‐1 and inhibits its activity.^79^ Very recently, the drug showed preclinical success in the treatment of liver cancer.^80^ Bindarit is a small‐molecule inhibitor of MCP‐1 that has completed phase II clinical trials for the treatment of diabetic nephropathy. The results indicated reduction in albuminuria upon 12‐week‐long administration of the drug.^81^ A MCP‐1 receptor antagonist, CCX140‐B, improved glycemic parameters in T2DM patients upon oral administration and provided renal protection in T2DM patients with nephropathy.^82^, ^83^ Antidiabetic drug Rosiglitazone (a TZD) was found to inhibit MCP‐1 in human mesangial cells subjected to mechanical stretch.^84^ Numerous statin drugs have also been found to decrease MCP‐1 levels and therefore can be considered as promising therapy for T2DM patients presenting hyperlipidemia.^85^, ^86^, ^87^ For example, a phase IV clinical trial to assess safety and efficacy of rosuvastatin monotherapy or in combination with ezetimibe is currently recruiting T2DM patients with hypercholesteremia (identifier # NCT03217409). Rosuvastatin lowers LDL cholesterol in diabetic patients and a combination of rosuvastatin and ibesartan (a MCP‐1 receptor antagonist) was shown to interfere with MCP‐1 signal transduction synergistically in vascular injury mice model.^88^ Similarly, low‐dose atorvastatin (Lipitor®) decreased MCP‐1 levels in T2DM patients with hyperlipidemia.^89^ Administration of n‐3 polyunsaturated fatty acid (PUFA) in T2DM patients resulted in significant decrease in MCP‐1 levels compared with placebo‐treated group, and therefore the anti‐inflammatory effects of n‐3 PUFAs were postulated to be mediated through modulation of MCP‐1.^90^

RBP‐4 is a plasma retinol transporter that shuttles retinol from liver to the peripheral tissues and is secreted by adipocytes.^91^ Elevated RBP‐4 levels were linked to higher body mass index (BMI) in diabetic and nondiabetic patients.^92^ However, this information is debatable because in at least two separate clinical trials, no significant difference in circulating RBP‐4 levels was obtained between normal and obese individuals.^65^, ^93^ In diabetes, higher circulating RBP‐4 level was positively correlated in impaired glucose tolerance, T2DM, and insulin resistance.^92^ The adipokine has been shown to reduce expression of glucose transporters in skeletal muscles, and thus decrease insulin sensitivity.^94^ Additionally, RBP4 triggers adipose tissue inflammation through activation of ATMs to secrete inflammatory cytokines.^95^ Expectedly, RBP4 knockdown has resulted in improved insulin sensitivity.^96^, ^97^ Rosuvastatin intake decreased serum levels of RBP‐4 in T2DM patients with hyperlipidemia.^98^ Another drug, fenretinide, a synthetic retinoid, was found to decrease RBP‐4 levels and improve insulin sensitivity and its long‐term therapy showed efficacy in alleviating obesity, insulin resistance, and hepatic steatosis in mice fed with high‐fat diet.^99^, ^100^ The drug is currently in phase II clinical trial to investigate its insulin sensitization efficacy in obese insulin‐resistant patients and in liver inflammation (identifier # NCT00546455). The drug is also being investigated in clinical trials as a chemotherapeutic agent for various cancers.^101^ A nonretinoid RBP‐4 antagonist, A1120, was shown to decrease serum RBP‐4 levels by 75% in mice.^102^

Chemerin is another novel adipokine that functions as a chemoattractant for monocytes and macrophages, promotes differentiation of preadipocytes, and is postulated to promulgate monocyte infiltration in adipose tissue of obese individuals, leading to low‐grade inflammation.^103^ The adipokine is involved in glucose and lipid homeostasis and high serum chemerin expression is found in inflammatory fluids.^103^ A positive cross talk between chemerin levels and impairment of glucose tolerance has been observed.^104^ In patients with chronic periodontitis and T2DM, chemerin levels in gingival crevicular fluid (GCF) were elevated compared with chronic periodontitis patients, suggesting that GCF chemerin can be a proinflammatory marker for diabetes and periodontal disease.^105^ Chemerin receptor, ChemR23 (also called CMKLR1) antagonist CCX832, was shown to reduce chemerin‐stimulated arterial contraction ex vivo and very recently shown to improve vascular function in obese diabetic mice.^12^, ^106^ However, another preclinical study demonstrated that CMKLR1 agonist rather than antagonist may be more beneficial for T2DM therapy because of chemerin agonist‐mediated increased insulin‐stimulated glucose uptake by adipocytes.^107^

ANGPTL2 is a recently identified adipokine that shares structural similarity with angiopoietin and found elevated in the serum of obese diabetic women.^108^ ANGPTL2 production was shown to be stimulated in human adipocyte culture exposed to stresses that mimic adipose tissue in obesity, and therefore the adipokine is assumed to promulgate adipose tissue inflammation and insulin resistance.^108^ Indeed, TNF‐α was found to induce ANGPTL2 expression in adipocytes in vitro.^109^ This was corroborated in a separate study where administration of recombinant ANGPTL2 to *db/db* mice induced proinflammatory gene production, increased macrophage subpopulation in adipose tissue that was primarily M1 polarized, and enhanced lipid accrual in the liver as well as fatty acid synthesis.^110^ ANGPTL2 knockdown mice showed similar insulin sensitivity and weight gain profile as wild‐type mice that underwent intermittent fasting, suggesting that reduction in ANGPTL2 levels is a promising strategy to counter development of insulin resistance and obesity.^111^ However, contradictory result was obtained by Kitazawa et al, who showed that exogenous administration of ANGPTL2 led to decrease in serum glucose, insulin, and fatty acids and concomitant increase in adiponectin and insulin sensitivity.^112^ Despite this, ANGPTL2 is mostly acknowledged as a proinflammatory adipokine involved in many chronic disorders such as diabetes and cancer.^113^ The adipokine is not currently under investigation in a clinical trial for treatment of T2DM.

Another novel adipokine, wingless‐related integration site‐1 (WNT‐1)‐inducible signaling pathway protein‐1 (WISP1), also known as CCN4, was shown to inhibit adipogenesis by preventing adipocyte differentiation and block PPAR‐γ transcriptional activity, thus believed to contribute toward the development of obesity.^114^ The proinflammatory potential of the adipokine was exhibited in vitro, when macrophages treated with WISP1 caused increased secretion of proinflammatory cytokines in a dose‐dependent fashion and polarized them to M1 phenotype.^115^ Serum concentration of the adipokine is elevated in obesity, irrespective of glycemia or insulin resistance status.^116^ This observation was reinforced in a different study, where WISP1 levels were found elevated in obese men irrespective of glycemic status compared with nonobese men.^117^ Additionally, the group showed that insulin signaling was impaired when hepatocytes were incubated with WISP1 due to inhibition of insulin receptor phosphorylation, other downstream regulators, and suppression of glycogenesis as well as gluconeogenesis. However, although the adipokine is positively correlated with visceral fat mass and is possibly a marker for insulin resistance, further studies are warranted to validate its role in T2DM onset or progression, before being considered for diabetes therapy.^115^

On the other hand, progranulin has an established role in obesity‐associated T2DM pathogenesis. Specifically, this cysteine‐rich protein decreases insulin‐mediated glucose uptake, disrupts insulin signal pathway, increases adipocyte autophagy, and promotes monocyte recruitment in adipose tissue as well as IL‐6 secretion from adipocytes.^118^, ^119^, ^120^ Elevated progranulin levels are present in obesity‐associated T2DM, nonalcoholic fatty liver disease, nephropathy, and retinopathy.^118^, ^121^ However, despite the proinflammatory role of progranulin in T2DM, it exhibits anti‐inflammatory function in various other disorders including arthritis, psoriasis, wound repair, and acute ischemia–reperfusion injury.^119^ Modulation of the adipokine is not being currently investigated for T2DM therapy in a clinical trial.

Lipocalin‐2 (LCN2) is an adipokine whose production is rapidly increased after differentiation of preadipocytes into mature adipocytes or under inflammatory conditions such as upon stimulation with lipopolysaccharide or IL‐1β.^122^ Moreover, LCN2 levels are reported to be high in obesity and directly proportional to fat mass, fasting blood glucose, and insulin resistance.^123^ Treatment with rosiglitazone decreased LCN2 serum concentrations and led to amelioration of insulin resistance in T2DM patients.^123^ In preclinical studies, LCN2 knock out in obese mice showed marked improvement in insulin sensitivity and glycemia compared with wild‐type mice and was attributed to suppression of production/activity of TNF‐α and 12‐lipoxygenase in the LCN2‐deficient mice.^122^ However, in patients with long‐term T2DM, serum LCN2 levels were found lower than healthy controls, following the similar trend as observed with anti‐inflammatory adipokines such as adiponectin.^124^ Administration of LCN2 shRNA using retroviral vector improved insulin activity in 3T3‐L1 adipocyte cells while exposure to LCN2 decreased insulin sensitivity in hepatocytes.^125^ Inhibition of LCN2 using monoclonal antibody has been explored for breast cancer therapy.^126^

Visfatin, an adipokine discovered in 2004, was found elevated in obesity in various clinical trials, but showed no correlation with insulin resistance, and its role in diabetes risk or progression is controversial.^127^, ^128^ Cardiotrophin‐1 is also an adipokine with divergent information about its role in regulation of glucose metabolism.^129^, ^130^, ^131^ Another adipokine, plasminogen activator inhibitor‐1 (PAI‐1), has been linked with T2DM but a systematic review comparing results of various clinical trials supports moderate association between them.^132^ Therefore, in light of the conflicting or moderate association between these adipokines and T2DM, they are not discussed in detail in this review. A summary of various adipokines that have central roles in mediating inflammation and strategies to mitigate the underlying inflammation in obesity‐associated T2DM is presented in Table 1.

**Table 1:** Adipokines involved in glucose homeostasis and notable therapies investigated for modulation of their activities for diabetes therapy

Adipokine	Pro/anti‐inflammatory	Drugs/formulations/strategies
Adiponectin	Anti‐inflammatory	Recombinant protein^33^, ^34^, ^35^, ^36^, ^37^, ^38^ AdipoRon (small‐molecule mimetic)^40^ Gene therapy^41^, ^42^, ^43^
ANGPTL2	Proinflammatory	Recombinant protein^110^
Apelin	Anti‐inflammatory	Recombinant protein^51^, ^54^ Acylated analogs of apelin‐13 amide^55^ pyr1‐apelin‐13 [CT]^56^ PEGylated apelin‐36^57^
Cartonectin	Anti‐inflammatory	Recombinant protein^64^ Exendin‐4^66^ Metformin [M]^67^
Chemerin	Proinflammatory	Receptor antagonist—CCX832^106^ CMKLR1 receptor agonist^107^
Leptin	Anti‐inflammatory	Metreleptin (Myalept™) [M] PEGylated leptin^47^ Gene therapy^48^, ^49^ Pluronic P85 conjugated leptin^50^
Lipocalin‐2	Anti‐inflammatory	Rosiglitazone [M]^123^ Gene therapy^125^
Monocyte chemoattractant protein‐1	Proinflammatory	NOX‐E36 (ematicap pegol) [CT]^78^ Small‐molecule inhibitor—Bindarit [CT]^81^ Receptor antagonists—CCX140‐B [CT], Ibesartan [M]^82^, ^83^, ^88^ Statins [M]^85^, ^86^, ^87^ Polyunsaturated fatty acids [CT]^90^
Retinol‐binding protein‐4	Proinflammatory	Retinoid‐based antagonist—Fenretinide [CT]^99^, ^100^ Nonretinoid antagonist—A1120^102^
Secreted frizzled protein‐5	Anti‐inflammatory	Liraglutide [M]^69^
Vaspin	Anti‐inflammatory	Recombinant protein^74^, ^75^

*Note:* FDA‐approved marketed drugs for treatment of T2DM or other indications and those in clinical trials are represented as [M] or [CT], respectively.

## 3 CYTOKINES AND THERAPIES THAT MODULATE THEIR ACTIVITY

Alongside adipokines, cytokines also significantly influence health and disease. Few important cytokines that promulgate diabetes or those with antidiabetic properties are discussed below. Alongside, notable drugs/formulations under investigation for modulation of their activities are reviewed.

### 3.1 Cytokines mediating insulin sensitization

An important cytokine possessing potent anti‐inflammatory property is IL‐10. Secreted primarily by T helper cells, monocytes, macrophages, and dendritic cells, it has a central role in curtailing inflammatory and autoimmune response of the host toward a pathogen, thus limiting inflammation‐mediated damage to the host.^133^, ^134^ Deficiency or impaired signaling/expression of IL‐10 can cause robust inflammatory response to a pathogen and development of inflammatory bowel disease or various autoimmune pathologies such as multiple sclerosis and T1DM.^134^, ^135^ T2DM patients also have significantly lower serum concentrations of IL‐10 compared with healthy individuals.^135^ However, serum IL‐10 levels were reported to be elevated in obese women but were lower in obese/nonobese women with metabolic syndrome.^136^ Various studies show that exogenous administration of recombinant IL‐10, IL‐10‐Fc protein, or plasmid DNA encoding IL‐10 can prevent onset of diabetes while treatment with antibody against IL‐10 receptor can induce diabetes in mice.^137^, ^138^, ^139^, ^140^, ^141^, ^142^, ^143^ One of the mechanisms of protection afforded by IL‐10 against insulin resistance in obesity is documented to be its ability to safeguard skeletal muscles from macrophage infiltration, thus prevent exposure to inflammatory conditions.^144^, ^145^ In T2DM, resistance or hyporesponsiveness to IL‐10 in macrophages exposed to high glucose may lead to deleterious glucose metabolism and techniques to overcome the hyporesponsiveness can be useful for treatment.^146^ To this end, a small‐molecule SHIP‐1 agonist, AQX‐MN100, that mimics anti‐inflammatory activity of IL‐10 was shown to bypass IL‐10 hyporesponsiveness and inhibit inflammation in cells exposed to high glucose.^146^ However, IL‐10‐based diabetes therapy is not being currently investigated in a clinical trial. A human recombinant IL‐10 formulation, Prevascar, was being developed by Renovo PLC for scar reduction but did not complete all clinical trials.^147^ Currently, a phase III clinical trial is underway for PEGylated human IL‐10 (AM0010, peigilodecakin) as a chemotherapeutic agent for metastatic pancreatic cancer treatment.^148^, ^149^

IL‐22 is another cytokine strongly associated with T2DM and prediabetes.^150^ In Han Chinese population, serum IL‐22 levels were significantly and progressively lower in participants with impaired fasting glucose and T2DM compared with normal participants.^150^ IL‐22 receptor‐deficient mice fed with high‐fat diet were susceptible to development of metabolic syndrome while exogenous administration of the cytokine to high‐fat‐diet‐fed obese *db/db* mice improved insulin sensitivity and glucose metabolism.^151^ Additionally, IL‐22 therapy was found to promote diabetic wound healing in vivo.^152^ McGuckin and coworkers demonstrated that IL‐22 therapy attenuated oxidative stress in pancreatic β cells, which suppressed inflammation and restored insulin sensitivity.^153^ However, other studies have shown contradictory results, stating that IL‐22 levels are elevated in T2DM.^154^, ^155^ The cytokine was termed as a “double edged sword” because IL‐22 also protected endothelial cells from glucose‐mediated injury.^154^ In a separate study, IL‐22 was shown to increase release of IL‐1β, a key proinflammatory cytokine, from macrophages.^156^ Currently, IL‐22‐based therapy is not being assessed for T2DM treatment, which is not surprising given the unclear role of the cytokine in the disease pathophysiology.

Another cytokine IL‐13 was shown to have a protective role in obesity and T2DM in preclinical trials. Although anti‐inflammatory, its expression seems to be induced in the adipose tissue by TNF‐α and IL‐1β.^157^ Despite being elevated in patients with insulin resistance, its levels did not show correlation with inflammation parameters such as TNF‐α and IL‐10.^158^ However, exogenous administration of IL‐13 improved insulin sensitivity in high‐fat‐diet C57BL/6 mice while IL‐13 gene overexpression improved glucose homeostasis by repressing adipose tissue inflammation and gluconeogenesis.^158^, ^159^ The expression of another cytokine, IL‐37, is positively correlated with insulin sensitivity and it was shown to inhibit activation of various proinflammatory signaling cycles, increase serum adiponectin levels, and decrease adipose tissue macrophages, thus protect against diet‐induced T2DM.^160^, ^161^ However, this anti‐inflammatory cytokine is not being actively investigated as a therapy in clinical trials currently. Other cytokines such as IL‐2 and IL‐4 have been primarily investigated as therapeutic agents in the context of T1DM, and therefore not being discussed in this review.^162^, ^163^, ^164^, ^165^

### 3.2 Cytokines mediating insulin resistance

**Figure 4:**
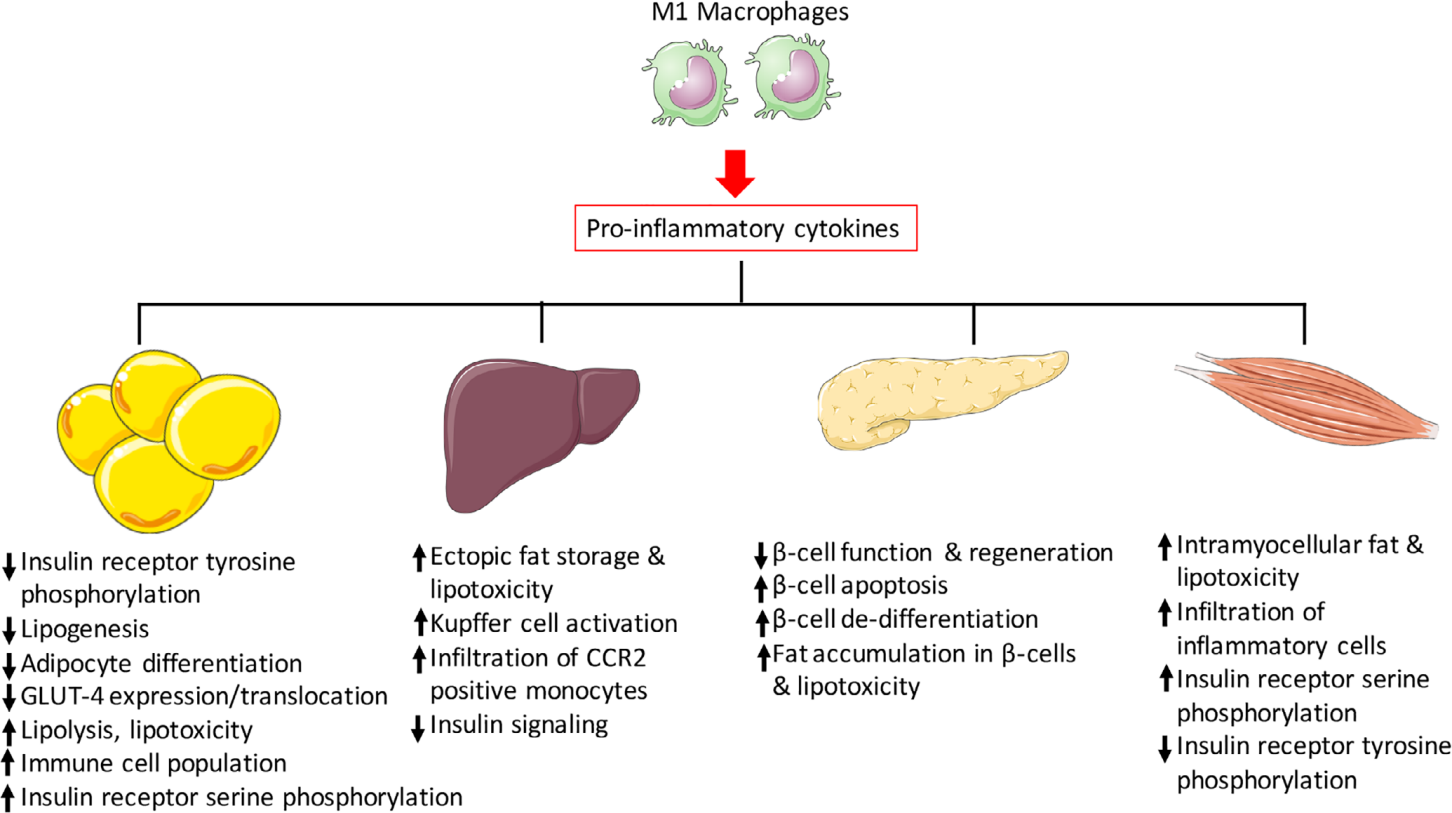
Mechanism of induction of peripheral insulin resistance due to chronic systemic presence of proinflammatory cytokines in insulin‐sensitive organs. For simplicity proinflammatory cytokine production from only M1 macrophages is shown in the figure. Various other immune cells including conventional dendritic cells, CD8^+^ T cells, and B cells present in the adipose tissue are also known to secrete proinflammatory cytokines

The pathophysiology of peripheral insulin resistance in insulin‐sensitive organs mediated by proinflammatory cytokines has been illustrated in Figure 4.

A proinflammatory cytokine with well‐established role in insulin resistance is TNF‐α.^166^, ^167^ Elevated amount of this cytokine is secreted from the adipose tissue in obesity while its knockout or receptor‐deficient animals are protected from insulin resistance development.^167^, ^168^, ^169^ The detrimental effect of TNF‐α on insulin signaling is caused by three different pathways: (a) reduction in insulin receptor tyrosine phosphorylation while increasing serine or threonine phosphorylation, (b) suppression of glucose transporter 4, and (c) increasing fatty acid lipolysis.^170^ Given the central role of TNF‐α in insulin resistance, its downregulation is a promising treatment strategy for T2DM therapy. Six different TNF‐α inhibitors have been clinically approved for the treatment of arthritis (rheumatoid, juvenile, psoriatic), plaque psoriasis, ankylosing spondylitis, ulcerative colitis, and/or Crohn's disease. These include adalumumab (Humira®), Adalimumab‐atto (Amjevita™), Certolizumab pegol (Cimzia®), Etanercept (Enbrel®), Golimumab (Simponi®), and Infliximab (Remicade®). Infliximab was also found to improve insulin sensitivity and enhance adiponectin production in patients with rheumatoid arthritis.^171^, ^172^ Similarly, in a study on 40 obese patients exhibiting impaired glucose homeostasis, prolonged etanercept administration improved fasting blood glucose and concentration of high‐molecular‐weight adiponectin.^173^ A pilot study carried out on newly diagnosed T1DM pediatric patients showed that etanercept lowers HbA1c levels and increases insulin production.^174^ However, in psoriatic patients with risk for T2DM, etanercept did not modulate insulin secretion or sensitivity.^175^ Similarly, adalimumab given to rheumatoid arthritis patients with insulin resistance did not improve insulin sensitivity, despite overall reduction in systemic inflammation.^176^ Several small‐molecule inhibitors of TNF‐α are also under investigation for inflammation modulation.^177^, ^178^ Another study on eight patients with rheumatoid arthritis or Crohn's disease with T2DM showed that treatment with anti‐TNF‐α significantly decreased fasting blood glucose levels, triglycerides, and in some cases HbA1c values compared with initial levels.^179^ In a preclinical study involving obese Wistar rats on high‐fat/high‐sucrose diet, blockade of TNF‐α receptor 1 using peptide Fc fusion protein for 4 weeks greatly improved insulin sensitivity compared with control.^180^ Likewise, silencing TNF‐α using siRNA encapsulated in glucan shells that primarily consist of β‐1,3‐d‐glucan, a ligand for the receptor of dectin‐1 expressed in macrophages, improved glucose tolerance in obese mice.^181^ Antidiabetic drug TZDs also block TNF‐α‐mediated impairment in insulin signaling.^182^ Concomitant reduction in IL‐6 and increase in adiponectin production can also be achieved with TZD therapy.^170^ Additionally, metformin was shown to reduce production and mRNA levels of TNF‐α in high‐fat‐diet‐fed obese mice by downregulating scavenger receptors CD36 and SR‐A in macrophages.^183^ Furthermore, nonsteroidal anti‐inflammatory drugs such as salicylates have shown usefulness in T2DM therapy through modulation of TNF‐α‐mediated inflammation. In a cohort of 286 patients treated for 48 weeks with salsalate (salicylate prodrug), a decrease in TNF‐α levels was observed along with lower HbA1c, fasting glucose, uric acid, and triglycerides with concomitant increase in adiponectin levels, mean HbA1c, and glycemia improvement compared with placebo group.^184^ Currently, the American Diabetes Association recommends the use of low‐dose aspirin for prevention of T1DM and T2DM patients with high risk of cardiovascular diseases.^185^

Proinflammatory cytokines in the IL‐1 family include IL‐1α and IL‐1β, of which the latter is strongly associated with development of both T1DM and T2DM.^186^, ^187^ Activation of nod‐like receptor protein 3 (NLRP3) inflammasome in the macrophages in response to metabolic stress factors recognized as “danger signals” such as increased fatty acids, ceramides, ROS, endoplasmic reticulum stress, extracellular ATP, urate, and islet amyloid polypeptide is known to trigger posttranslational modification and subsequent release of IL‐1β and IL‐18.^188^, ^189^, ^190^ IL‐1β is implicated in pancreatic β‐cell functional impairment, apoptosis, and consequent decrease in β‐cell mass.^186^, ^191^, ^192^ Additionally, high blood glucose concentration leads to stimulation of its own release from the beta cells and recruitment of macrophages that further increase IL‐1β levels.^186^, ^191^, ^192^ Several IL‐1β knockdown strategies including IL‐1β receptor antagonist have been investigated for the treatment of T2DM. Out of these, the most notable one includes Anakinra (Kineret®), a marketed IL‐1β receptor antagonist used for the treatment of rheumatoid arthritis. A multicenter, randomized phase IV TRACK [Treatment of Rheumatoid Arthritis and Comorbidities with Kineret (anakinra)] study was being conducted to determine the effect of anakinra on lowering HbA1c levels in rheumatoid arthritis patients with T2DM (identifier # NCT02236481), but was terminated by the sponsor for early benefits. It was reported that treatment with anakinra on two patients with rheumatoid arthritis and T2DM was beneficial in reaching the therapeutic targets for both diseases.^193^ In patients with gout and T2DM, Anakinra treatment resulted in significant improvement in joint functions and glycemic control.^194^ A week‐long daily administration of 100 mg anakinra in insulin‐resistant T1DM patients resulted in significant improvement in insulin sensitivity for 4 weeks after treatment.^195^ Other clinically approved IL‐1β‐based treatments for crypopyrin‐associated periodic syndromes include canakinumab (Ilaris®, IL‐1β antagonist) and rilonacept (Arcalyst®, soluble decoy IL‐1 receptor).^196^ In the context of diabetes, treatment with canakinumab did not prevent progression from prediabetes to new onset diabetes nor decrease HbA1c and glucose levels over a long term.^197^ In this clinical trial, decrease in HbA1c was observed only during the first 6–9 months of treatment but not consistently over a longer period. Indeed, a decrease in HbA1c was noted in an earlier trial in T2DM patients on metformin upon using canakinumab for 4 months.^198^ On the other hand, rilonacept has been tested for T1DM therapy in a phase I study and showed good tolerance, but posited “unlikely to be efficacious as a single agent in preserving beta‐cell function.”^199^ Gevokizumab is another IL‐1β antagonist that enhanced insulin sensitivity and decreased inflammation in T2DM patients.^200^ A high‐affinity neutralizing antibody against IL‐1β, LY2189102, was also found to decrease inflammation and HbA1c.^201^ Similarly, a monoclonal antibody against IL‐1 β, XOMA052, was shown to prevent high‐fat‐diet‐induced impaired glucose tolerance and insulin production while decreasing β‐cell death in obese mice.^202^ Furthermore, Zha et al have developed an epitope peptide vaccine targeting IL‐1β with polylactic acid microsphere adjuvant to neutralize IL‐1β activity.^203^ When administered to diabetic KK‐A^y^ mice, the formulation was able to enhance glucose tolerance, insulin sensitivity while decrease free fatty acids in serum, cholesterol, β‐cell death, and IL‐1β production. Likewise, a different peptide antagonist of IL‐1β, SER140, was found to preserve β‐cell mass and delay the diabetes development in NOD mice.^204^

Another interleukin IL‐6, traditionally considered proinflammatory, is now believed to display both inflammatory and protective roles against inflammation, and its contribution toward T1DM or T2DM development remains debatable.^205^ However, Tocilizumab, an anti‐IL‐6 receptor antibody, was found to decrease HbA1c levels in patients to a greater extent than TNF‐α inhibitors.^206^ Similarly, there is conflicting evidence about the role of IL‐18 in T2DM.^207^, ^208^ While the serum levels of IL‐18 are elevated in obese and T2DM patients, therefore thought to mediate insulin resistance, studies have also shown glucose‐lowering potential of IL‐18.^208^, ^209^, ^210^ Other interleukins such as IL‐12 and IL‐17 have been reported elevated in T2DM.^211^, ^212^ However, in a preclinical study, IL‐17 was shown to negatively correlate with glucose homeostasis and adipogenesis.^213^ An antibody, ustekinumab (Stelara®), that binds to IL‐12 and IL‐23 and approved for the treatment of plaque psoriasis is currently undergoing phase II/III clinical trials for the treatment of T1DM (identifier # NCT03941132). Owing to contradictory evidence of these cytokines about their role in T2DM development, they are not discussed in detail in this review.

Resistin is another hormone secreted by adipocytes in mice but primarily expressed by macrophages and peripheral blood mononuclear cells (PBMCs) in humans.^214^ As the name suggests, the hormone is strongly associated with inflammation and insulin resistance. Heightened serum levels of resistin are present in diet‐induced obese mice and in diabetic mice.^215^ In humans, a significant positive correlation between fasting serum resistin levels, C‐reactive protein (inflammation biomarker), insulin resistance, fasting blood glucose levels, body mass index, and triglycerides was found and it was associated with T2DM‐induced retinopathy.^216^ The production of human resistin from macrophages and PBMCs is escalated upon treatment with lipopolysaccharide and proinflammatory cytokines while on the other hand, presence of human resistin also induces production of proinflammatory adipocytokines, leading to a vicious cycle of chronic inflammation.^214^ However, conflicting reports showing no positive correlation between serum resistin levels and insulin resistance markers as well as adiposity have also been documented in T2DM patients, probably making its role in human diabetes contentious.^217^ As a drug target, several antagonists of resistin have been developed, such as a mutant version of resistin (C6A), which was shown to improve glucose tolerance and insulin sensitivity in high‐fat‐diet‐fed insulin‐resistant mice.^218^ A resistin binding peptide was developed by Gu et al and shown to negate the function of resistin in vitro.^219^ In overweight women with polycystic ovarian disease, rosiglitazone decreased resistin levels and increased adiponectin levels after 4 months of treatment.^220^ A 12‐week‐long treatment comparing efficacy of pioglitazone and metformin in decreasing serum resistin levels, fasting blood glucose, and insulin concentration was completed in T2DM children, but results of the clinical trial have not been posted (identifier # NCT01396564). Antibodies against resistin and its putative receptors such as toll‐like receptor 4 and adenylyl cyclase‐associated protein 1 can be candidate therapeutic agents to antagonize resistin function.^221^, ^222^ Antioxidants may also be useful in lowering resistin levels. A clinical trial studying the effect of intake of alpha‐lipoic acid on serum resistin levels in T2DM patients with chronic periodontitis demonstrated that systemic administration of alpha‐lipoic acid as an adjunct therapy to scaling and root planning significantly decreased serum resistin and HbA1c in the patients.^223^

Transforming growth factor β (TGF‐β) secreted by macrophages and monocytes drives renal cell hypertrophy and buildup of extracellular matrix, and therefore plays a central role in diabetic nephropathy, a long‐term complication of T2DM.^224^, ^225^, ^226^, ^227^ The cytokine levels are elevated in serum and urine of T2DM individuals with diabetes nephropathy.^228^ An increased expression of the cytokine was also observed in adipose tissues of obese mice and attributed to upregulation of TNF‐α levels.^229^ Blockade of TGF‐β signaling with anti‐TGF‐β1 antibody protected mice from diet‐induced obesity and diabetes.^230^ An antibody against TGF‐β, fresolimumab (GC 1008), is being investigated for the treatment of glomerulosclerosis, systemic sclerosis, pulmonary fibrosis, and various cancer, but not in particular context of insulin resistance.^230^, ^231^, ^232^

CXCL5 secreted by white adipose tissue macrophages is also known to promote obesity‐induced insulin resistance and various other obesity‐related inflammatory disorders including atherosclerosis, retinopathy, and ulcerative colitis, among others.^233^, ^234^ Its expression is regulated by TNF‐α and significantly increased in obese subjects compared with lean individuals.^233^, ^235^ CXCL5 inhibition in insulin‐resistant obese mice with anti‐CXCL5 antibody or its specific receptor antagonist significantly improved glucose tolerance and insulin sensitivity.^233^, ^234^ Diabetes treatment based on CXCL5 neutralization is not being currently investigated in a clinical trial.

Macrophage migration inhibitory factor (MIF) is a proinflammatory cytokine secreted from a variety of cells including T‐lymphocytes, macrophages/monocytes, eosinophils, neutrophils, and endothelial cells, among others.^236^ Serum MIF level is elevated in prediabetes, T2DM, and gestational diabetes and a positive correlation was found between serum MIF concentration and impaired glucose tolerance, insulin resistance, BMI, and visceral fat mass in various clinical trials.^237^, ^238^ Although originally recognized as macrophage migration inhibitor, the cytokine is known to induce secretion of proinflammatory cytokines including TNF‐α, IL‐1, 6, 8, and 12, recruit various inflammatory cells, and upregulate expression of adhesion molecules on monocytes and endothelial cells.^236^ Several small‐molecule inhibitors of MIF have been investigated for cancer therapy, inflammatory, and immune disorders.^239^, ^240^, ^241^ An oral small‐molecule inhibitor of MIF, CSPI‐1306, was found to reduce blood glucose, TNF‐α, and IL‐6 levels in type 2 diabetic outbred Institute of Cancer Research (ICR) mice.^242^ Administration of another small‐molecule inhibitor of MIF, ISO‐1, to diabetic *db/db* mice reduced blood glucose and diabetic nephropathy conditions such as albuminuria, activation of macrophages, and extracellular matrix growth in diabetic kidney.^243^ Inhibition of MIF receptor, CD74, using specific shRNA decreased high‐fat‐diet‐induced polarization of macrophages to M1 phenotype and insulin resistance in mice.^244^ Additionally, RNAi therapy to inhibit cyclooxygenase‐2 (COX‐2) was found to reduce MIF levels in adipocytes exposed to high fat.^244^

A summary of various cytokines that have central roles in mediating inflammation and strategies to mitigate the underlying inflammation in obesity‐associated T2DM are presented in Table 2.

**Table 2:** Cytokines involved in glucose homeostasis and notable therapies investigated for modulation of their activities for diabetes therapy

Cytokine	Pro/anti‐inflammatory	Drugs/formulations/strategies
CXCL5	Proinflammatory	Antibodies^233^, ^234^
Interleukin‐1β	Proinflammatory	Inhibitors—Anakinra [M], Canakinumab [M], Rilonacept [M], Gevokizumab [CT], LY2189102 [CT]^193^, ^194^, ^195^, ^197^, ^198^, ^199^, ^200^ Antibodies^201^, ^202^ Peptide antagonists^203^, ^204^
Interleukins 10, 13, 22, 37	Anti‐inflammatory	Recombinant protein^138^ Gene therapy^139^, ^140^, ^141^, ^142^ IL‐10‐Fc protein^143^ Small‐molecule mimetic—AQX‐MN100^146^
Macrophage inhibitory factor	Proinflammatory	Small‐molecule inhibitors^242^, ^243^ Gene therapy^244^
Transforming growth factor‐β	Proinflammatory	Antibody^230^
Tumor necrosis factor‐α	Proinflammatory	Inhibitors—Etanercept [M], Infliximab [M]^171^, ^172^, ^173^, ^174^ Small‐molecule inhibitors^177^, ^178^ Receptor blockade—Fc fusion protein^180^ Gene therapy^181^ Thiazolidinediones [M]^170^, ^182^ Metformin [M]^183^ Salicylates [M]^184^
Resistin	Proinflammatory	Mutant resistin (C6A)^218^ Rosiglitazone [M]^220^ Antibodies against resistin and its receptor^219^, ^221^, ^222^ Alpha lipoic acid [CT]^223^

*Note:* FDA‐approved marketed drugs for treatment of T2DM or other indications and those in clinical trials are represented as [M] or [CT], respectively.

Taken together, a strong correlation exists between expression of several adipocytokines and systemic inflammation leading to insulin resistance in obesity‐associated T2DM. Therefore, these molecules present an appealing class of compounds that can be modulated to obtain significant therapeutic benefit in T2DM treatment. Consequently, several strategies are being developed to mitigate the underlying inflammation in obesity‐associated T2DM.

## 4 CONCLUSIONS

The adipose tissue is now considered an endocrine organ that secretes various molecules with paracrine or endocrine functions. Among them, adipocytokines secreted from adipocytes or adipose tissue immune cells are vital inflammatory modulators that regulate various physiological processes including metabolism and immunologic response. Increase in adiposity alters adipose tissue microenvironment and skews resident cells toward proinflammatory phenotype that significantly impairs glucose homeostasis and leads to development of obesity and T2DM. Hence, remodeling of adipose tissue inflammasome through immunoneutralization, small‐molecule inhibitors/agonists, exogenous administration of adipocytokines or mimetics, gene therapy, and novel formulations can bring significant benefit in T2DM therapy. Further development of these treatment strategies is essential to treat the underlying cause of insulin resistance in obesity‐associated T2DM, and thereby improve diabetes treatment outcome. The diversity of adipocytokines and immune cells involved in type 2 diabetes maintenance/progression opens prospect of disease modulation through various novel approaches. However, many adipocytokines are multifaceted and their physiological role is not well understood. Targeting adipose tissue inflammation for successful therapies would therefore first entail better understanding of the functions and crosstalk between various adipocytokines in the context of diabetes. Additionally, alleviation of inflammation in other tissues or organs that undergo fat cell and/or proinflammatory immune cell infiltration such as the muscle, liver, and pancreas should also be considered for developing effective T2DM therapy.

## CONFLICT OF INTEREST

The authors declare no conflict of interest.

## AUTHOR CONTRIBUTIONS

All authors have read and approved the final manuscript. A.B. conceived the review topic and wrote the manuscript. J.S. critically reviewed the manuscript.
